# Di­bromido­bis­(2-methyl-1*H*-benzimidazole-κ*N* ^3^)cadmium

**DOI:** 10.1107/S1600536813024549

**Published:** 2013-09-07

**Authors:** Bao-Cheng Liu, Yan-Ling Jin, Fa-Qian Liu

**Affiliations:** a Key Laboratory of Advanced Materials, Qingdao University of Science and Technology, Qingdao 266042, People’s Republic of China

## Abstract

In the title compound, [CdBr_2_(C_8_H_8_N_2_)_2_], the Cd^II^ atom has a distorted tetra­hedral coordination formed by the two imino N atoms of two 2-methyl­benzimidazole ligands and two terminal bromide ligands. The Cd^II^ atom is slightly out of the benzimidazole planes by 0.320 (3) and 0.210 (3) Å. The dihedral angle between the benzimidazole planes is 71.6 (2)°. In the crystal, mol­ecules are linked by N—H⋯Br hydrogen bonds into puckered layers parallel to (001).

## Related literature

For background to benzimidazole, see: Roderick *et al.* (1972 ▶). For related crystal structures, see: Barros-García *et al.* (2005 ▶); Wang *et al.* (2010 ▶); Yang *et al.* (2011 ▶).
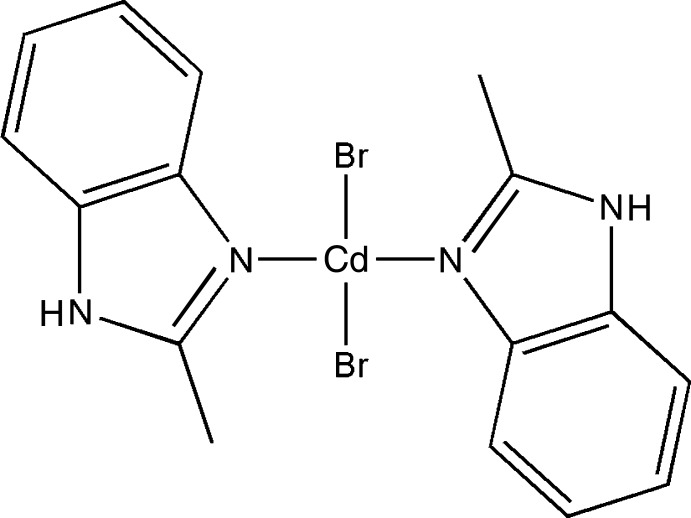


## Experimental

### 

#### Crystal data

[CdBr_2_(C_8_H_8_N_2_)_2_]
*M* *_r_* = 536.54
Monoclinic, 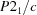

*a* = 10.007 (9) Å
*b* = 14.747 (12) Å
*c* = 12.399 (11) Å
β = 93.088 (14)°
*V* = 1827 (3) Å^3^
*Z* = 4
Mo *K*α radiation
μ = 5.57 mm^−1^
*T* = 296 K
0.22 × 0.18 × 0.16 mm

#### Data collection

Rigaku R-AXIS Spider diffractometer
Absorption correction: multi-scan (*ABSCOR*; Higashi, 1995 ▶) *T*_min_ = 0.374, *T*_max_ = 0.469
9698 measured reflections
3585 independent reflections
2748 reflections with *I* > 2σ(*I*)
*R*_int_ = 0.032

#### Refinement

*R*[*F* ^2^ > 2σ(*F* ^2^)] = 0.033
*wR*(*F* ^2^) = 0.077
*S* = 1.00
3585 reflections
210 parameters
H-atom parameters constrained
Δρ_max_ = 0.39 e Å^−3^
Δρ_min_ = −1.02 e Å^−3^

### 

Data collection: *RAPID-AUTO* (Rigaku, 2004 ▶); cell refinement: *RAPID-AUTO*; data reduction: *RAPID-AUTO*; program(s) used to solve structure: *SHELXTL* (Sheldrick, 2008 ▶); program(s) used to refine structure: *SHELXTL* (Sheldrick, 2008 ▶); molecular graphics: *SHELXTL*; software used to prepare material for publication: *SHELXTL*.

## Supplementary Material

Crystal structure: contains datablock(s) global, I. DOI: 10.1107/S1600536813024549/kq2008sup1.cif

Structure factors: contains datablock(s) I. DOI: 10.1107/S1600536813024549/kq2008Isup2.hkl

Supplementary material file. DOI: 10.1107/S1600536813024549/kq2008Isup3.cdx

Additional supplementary materials:  crystallographic information; 3D view; checkCIF report

## Figures and Tables

**Table 1:** Hydrogen-bond geometry (Å, °)

*D*—H⋯*A*	*D*—H	H⋯*A*	*D*⋯*A*	*D*—H⋯*A*
N2—H2⋯Br1^i^	0.86	2.88	3.495 (4)	130
N4—H4⋯Br2^ii^	0.86	2.77	3.563 (4)	155

Symmetry codes: (i) 
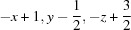
; (ii) 
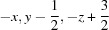
.

**Fig. 1.**
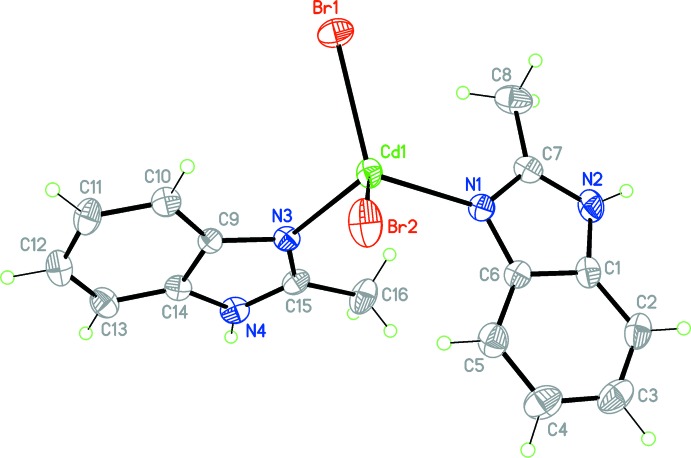
Molecular structure of the title compound. Displacement ellipsoids are shown at the 40% probability level. H atoms are presented as small spheres of arbitrary radius.

**Fig. 2.**
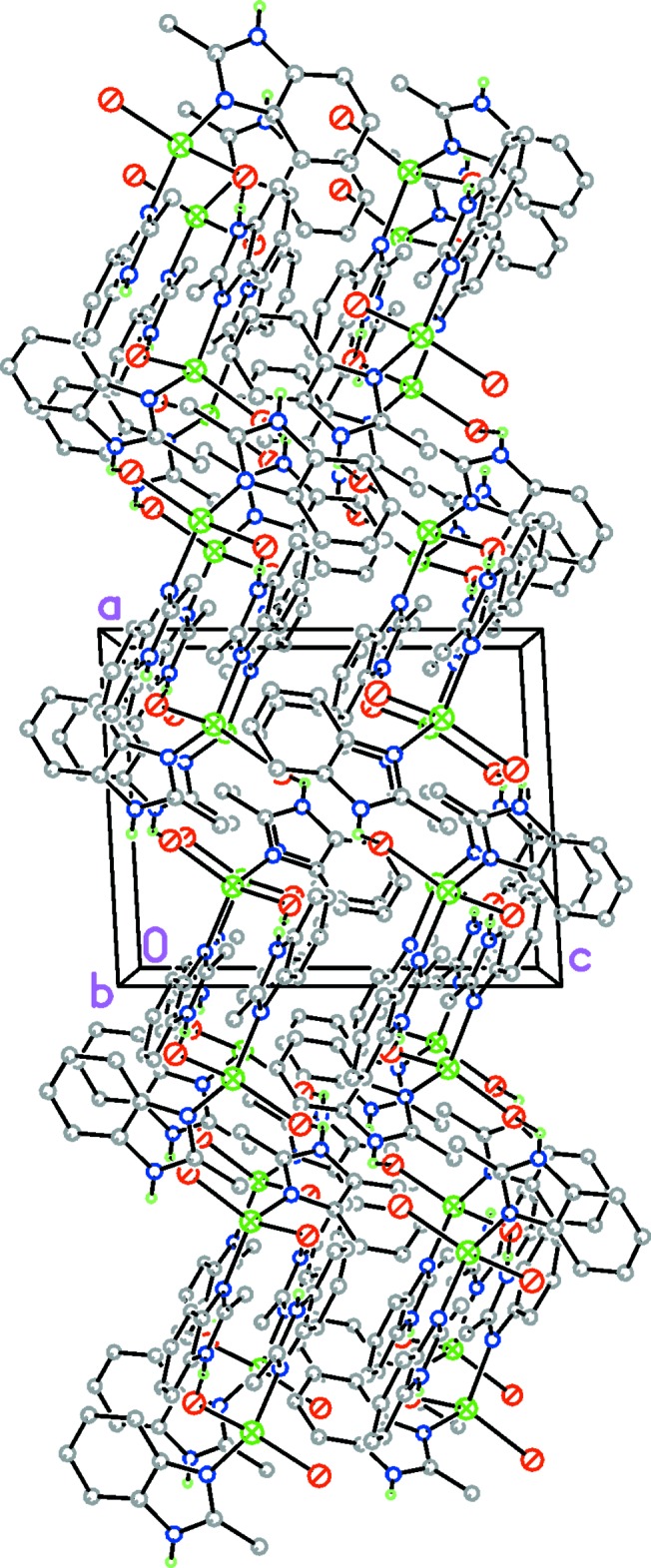
Crystal packing of the title compound along the b axis demonstrating the puckered layers parallel to (001). Dashed lines indicate the intermolecular hydrogen bonds.

## supplementary crystallographic information

### 1. Comment

Benzimidazole and its derivatives have attracted interest because their biological activities as well as their abilities to bind different metal ions (Roderick *et al.*, 1972). In this paper, we describe the synthesis and structure of dibromo-bis(2-methylbenzimidazole)-cadmium(II).

In the title compound, C_16_H_16_CdBr_2_N_4_, the cadmium atom has a distorted tetrahedral coordination formed by the two imino-nitrogen atoms of two 2-methyl-benzimidazole ligands and two terminal bromide ligands (Figure 1). The similar geometry was previously found in the related compounds – Cd(Cl)_2_(*N*-(5,6-dihydro-4*H*- 1,3-thiazin-2-yl)-2-aminobenzimidazole)_2_ (Barros-García *et al.*, 2005), Cd(Cl)_2_(2-(2-furyl)-1-(2-furylmethyl)-1*H*-benzimidazole)_2_ (Wang *et al.*, 2010), and Cd(I)_2_(2-(2-furyl)-1-(2-furylmethyl)- 1*H*-enzimidazole)_2_ (Yang *et al.*, 2011). The cadmium atom is slightly out of the two benzimidazole planes by 0.320 (3) and 0.210 (3) Å, respectively. The dihedral angle between the two benzimidazole planes is 71.6 (2)°. The mean values of Cd—Br and Cd—N bond lengths are 2.562 (2) and 2.251 (3) Å, respectively. The N—Cd—Br bond angles range from 105.40 (10) to 117.76 (9)°.

In the crystal, the molecules of the title compound are linked by intermolecular N2—H2···Br1^i^ and N4—H4···Br2^ii^ hydrogen bonds (Table 1) into puckered layers parallel to (001) (Figure 2). Symmetry codes: (i) –*x*+1, *y*–1/2, –*z*+3/2; (ii) –*x*, *y*–1/2, –*z*+3/2.

### 2. Experimental

The ligand 2-methyl-benzimidazole (0.02 mmol) in ethanol (10 mL) was added dropwise to a ethanol (10 mL) of CdBr_2 _(0.01 mmol). The resulting solution was allowed to stand at room temperature. After one week colorless crystals with good quality were obtained from the filtrate and dried in air. Analysis, calculated for C_16_H_16_Br_2_CdN_4_: C 35.82, H 3.01, N 10.44%; Found: C 35.68, H 3.02, N 10.47%.

### 3. Refinement

All hydrogen atoms were placed in calculated positions with N—H = 0.86 Å and C—H = 0.93 (aryl-H) and 0.96 (methyl-H) Å and refined in the riding model with fixed isotropic displacement parameters [*U*_iso_(H) = 1.2*U*_eq_(N or C)].

### Crystal data

**Table:**

[CdBr_2_(C_8_H_8_N_2_)_2_]	*F*(000) = 1032
*M**_r_* = 536.54	*D*_x_ = 1.951 Mg m^−^^3^
Monoclinic, *P*2_1_/*c*	Mo *K*α radiation, λ = 0.71073 Å
Hall symbol: -P 2ybc	Cell parameters from 3275 reflections
*a* = 10.007 (9) Å	θ = 2.5–26.6°
*b* = 14.747 (12) Å	µ = 5.57 mm^−^^1^
*c* = 12.399 (11) Å	*T* = 296 K
β = 93.088 (14)°	Block, colorless
*V* = 1827 (3) Å^3^	0.22 × 0.18 × 0.16 mm
*Z* = 4	

### Data collection

**Table:**

Rigaku R-AXIS Spider diffractometer	2748 reflections with *I* > 2σ(*I*)
Radiation source: fine-focus sealed tube	*R*_int_ = 0.032
Graphite monochromator	θ_max_ = 26.0°, θ_min_ = 2.0°
ω scans	*h* = −12→10
Absorption correction: multi-scan (*ABSCOR*; Higashi, 1995)	*k* = −18→14
*T*_min_ = 0.374, *T*_max_ = 0.469	*l* = −15→14
9698 measured reflections	13 standard reflections every 0 reflections
3585 independent reflections	intensity decay: none

### Refinement

**Table:**

Refinement on *F*^2^	Primary atom site location: structure-invariant direct methods
Least-squares matrix: full	Secondary atom site location: difference Fourier map
*R*[*F*^2^ > 2σ(*F*^2^)] = 0.033	Hydrogen site location: inferred from neighbouring sites
*wR*(*F*^2^) = 0.077	H-atom parameters constrained
*S* = 1.00	*w* = 1/[σ^2^(*F*_o_^2^) + (0.0407*P*)^2^] where *P* = (*F*_o_^2^ + 2*F*_c_^2^)/3
3585 reflections	(Δ/σ)_max_ = 0.001
210 parameters	Δρ_max_ = 0.39 e Å^−^^3^
0 restraints	Δρ_min_ = −1.02 e Å^−^^3^

### Special details

**Table:**

Geometry. All e.s.d.'s (except the e.s.d. in the dihedral angle between two l.s. planes) are estimated using the full covariance matrix. The cell e.s.d.'s are taken into account individually in the estimation of e.s.d.'s in distances, angles and torsion angles; correlations between e.s.d.'s in cell parameters are only used when they are defined by crystal symmetry. An approximate (isotropic) treatment of cell e.s.d.'s is used for estimating e.s.d.'s involving l.s. planes.
Refinement. Refinement of *F*^2^ against ALL reflections. The weighted *R*-factor *wR* and goodness of fit *S* are based on *F*^2^, conventional *R*-factors *R* are based on *F*, with *F* set to zero for negative *F*^2^. The threshold expression of *F*^2^ > σ(*F*^2^) is used only for calculating *R*-factors(gt) *etc*. and is not relevant to the choice of reflections for refinement. *R*-factors based on *F*^2^ are statistically about twice as large as those based on *F*, and *R*- factors based on ALL data will be even larger.

### Fractional atomic coordinates and isotropic or equivalent isotropic displacement parameters (Å^2^)

**Table:**

	*x*	*y*	*z*	*U*_iso_*/*U*_eq_	
N1	0.3592 (3)	0.96335 (19)	0.8577 (2)	0.0362 (7)	
N2	0.5028 (4)	0.8661 (2)	0.9341 (3)	0.0485 (9)	
H2	0.5712	0.8311	0.9428	0.058*	
N3	0.0723 (3)	1.01679 (18)	0.6820 (2)	0.0339 (7)	
N4	−0.1049 (3)	0.93220 (19)	0.6357 (2)	0.0417 (8)	
H4	−0.1554	0.8851	0.6290	0.050*	
Br1	0.40130 (5)	1.14156 (3)	0.60773 (4)	0.06071 (16)	
Br2	0.20546 (5)	1.20099 (3)	0.88850 (4)	0.06367 (17)	
C1	0.4087 (4)	0.8806 (2)	1.0086 (3)	0.0409 (9)	
C2	0.3939 (5)	0.8457 (3)	1.1109 (3)	0.0531 (12)	
H2A	0.4561	0.8060	1.1434	0.064*	
C3	0.2816 (6)	0.8730 (3)	1.1622 (4)	0.0626 (14)	
H3	0.2681	0.8511	1.2311	0.075*	
C4	0.1873 (6)	0.9326 (3)	1.1139 (4)	0.0633 (13)	
H4A	0.1119	0.9482	1.1505	0.076*	
C5	0.2045 (5)	0.9687 (3)	1.0127 (3)	0.0535 (11)	
H5	0.1432	1.0094	0.9812	0.064*	
C6	0.3175 (4)	0.9417 (2)	0.9597 (3)	0.0368 (9)	
C7	0.4708 (4)	0.9156 (2)	0.8454 (3)	0.0408 (9)	
C8	0.5489 (5)	0.9139 (3)	0.7478 (4)	0.0620 (13)	
H8A	0.4935	0.9338	0.6867	0.093*	
H8B	0.5793	0.8532	0.7355	0.093*	
H8C	0.6246	0.9536	0.7578	0.093*	
C9	−0.0236 (4)	1.0707 (2)	0.6250 (3)	0.0332 (8)	
C10	−0.0180 (4)	1.1618 (2)	0.5961 (3)	0.0420 (9)	
H10	0.0571	1.1972	0.6131	0.050*	
C11	−0.1303 (5)	1.1972 (3)	0.5406 (3)	0.0502 (11)	
H11	−0.1297	1.2575	0.5187	0.060*	
C12	−0.2441 (5)	1.1449 (3)	0.5168 (3)	0.0531 (11)	
H12	−0.3184	1.1720	0.4820	0.064*	
C13	−0.2499 (4)	1.0548 (3)	0.5431 (3)	0.0482 (10)	
H13	−0.3255	1.0199	0.5259	0.058*	
C14	−0.1372 (4)	1.0184 (2)	0.5966 (3)	0.0364 (9)	
C15	0.0180 (4)	0.9343 (2)	0.6856 (3)	0.0386 (9)	
C16	0.0818 (5)	0.8531 (2)	0.7384 (3)	0.0577 (13)	
H16A	0.1749	0.8514	0.7233	0.087*	
H16B	0.0384	0.7992	0.7108	0.087*	
H16C	0.0733	0.8566	0.8150	0.087*	
Cd1	0.26490 (3)	1.074421 (18)	0.75481 (2)	0.03930 (11)	

### Atomic displacement parameters (Å^2^)

**Table:**

	*U*^11^	*U*^22^	*U*^33^	*U*^12^	*U*^13^	*U*^23^
N1	0.039 (2)	0.0255 (15)	0.0436 (18)	0.0043 (14)	0.0033 (15)	0.0002 (14)
N2	0.044 (2)	0.0340 (18)	0.066 (2)	0.0133 (15)	−0.0060 (18)	0.0078 (17)
N3	0.0365 (18)	0.0242 (15)	0.0410 (16)	−0.0068 (13)	0.0015 (14)	−0.0021 (13)
N4	0.048 (2)	0.0289 (17)	0.0482 (19)	−0.0128 (14)	0.0016 (16)	−0.0055 (14)
Br1	0.0653 (3)	0.0460 (3)	0.0713 (3)	−0.0150 (2)	0.0077 (2)	0.0149 (2)
Br2	0.0746 (4)	0.0398 (3)	0.0745 (3)	0.0205 (2)	−0.0163 (3)	−0.0179 (2)
C1	0.044 (2)	0.027 (2)	0.050 (2)	−0.0007 (17)	−0.010 (2)	0.0000 (18)
C2	0.066 (3)	0.039 (2)	0.053 (3)	−0.008 (2)	−0.017 (2)	0.006 (2)
C3	0.089 (4)	0.058 (3)	0.041 (2)	−0.018 (3)	0.003 (3)	−0.003 (2)
C4	0.082 (4)	0.057 (3)	0.052 (3)	0.005 (3)	0.016 (3)	−0.005 (2)
C5	0.058 (3)	0.049 (3)	0.053 (3)	0.016 (2)	0.002 (2)	−0.003 (2)
C6	0.043 (2)	0.0256 (19)	0.041 (2)	−0.0001 (16)	0.0017 (18)	−0.0006 (16)
C7	0.039 (2)	0.030 (2)	0.053 (2)	0.0008 (17)	0.0057 (19)	−0.0037 (18)
C8	0.057 (3)	0.050 (3)	0.081 (3)	0.006 (2)	0.027 (3)	−0.005 (2)
C9	0.037 (2)	0.030 (2)	0.0328 (19)	0.0039 (16)	0.0046 (16)	−0.0025 (16)
C10	0.046 (3)	0.033 (2)	0.048 (2)	−0.0044 (18)	0.0004 (19)	−0.0034 (18)
C11	0.068 (3)	0.029 (2)	0.053 (2)	0.013 (2)	−0.003 (2)	−0.0004 (19)
C12	0.051 (3)	0.054 (3)	0.053 (3)	0.013 (2)	−0.010 (2)	−0.006 (2)
C13	0.041 (3)	0.053 (3)	0.050 (2)	−0.004 (2)	−0.005 (2)	−0.011 (2)
C14	0.041 (2)	0.035 (2)	0.0341 (19)	−0.0006 (17)	0.0046 (17)	−0.0080 (16)
C15	0.051 (3)	0.028 (2)	0.038 (2)	−0.0037 (17)	0.0054 (19)	0.0007 (16)
C16	0.082 (4)	0.030 (2)	0.059 (3)	−0.008 (2)	−0.014 (2)	0.011 (2)
Cd1	0.04113 (19)	0.02686 (16)	0.04918 (18)	0.00061 (12)	−0.00430 (13)	0.00266 (12)

### Geometric parameters (Å, º)

**Table:**

N1—C7	1.336 (5)	C4—H4A	0.9300
N1—C6	1.390 (5)	C5—C6	1.396 (6)
N1—Cd1	2.252 (3)	C5—H5	0.9300
N2—C7	1.345 (5)	C7—C8	1.475 (6)
N2—C1	1.370 (5)	C8—H8A	0.9600
N2—H2	0.8600	C8—H8B	0.9600
N3—C15	1.334 (4)	C8—H8C	0.9600
N3—C9	1.407 (5)	C9—C10	1.393 (5)
N3—Cd1	2.250 (3)	C9—C14	1.403 (5)
N4—C15	1.347 (5)	C10—C11	1.387 (6)
N4—C14	1.392 (5)	C10—H10	0.9300
N4—H4	0.8600	C11—C12	1.394 (6)
Br1—Cd1	2.5372 (15)	C11—H11	0.9300
Br2—Cd1	2.5869 (15)	C12—C13	1.370 (6)
C1—C2	1.385 (6)	C12—H12	0.9300
C1—C6	1.398 (5)	C13—C14	1.386 (6)
C2—C3	1.381 (7)	C13—H13	0.9300
C2—H2A	0.9300	C15—C16	1.492 (5)
C3—C4	1.401 (7)	C16—H16A	0.9600
C3—H3	0.9300	C16—H16B	0.9600
C4—C5	1.381 (6)	C16—H16C	0.9600
			
C7—N1—C6	106.1 (3)	C7—C8—H8C	109.5
C7—N1—Cd1	130.2 (3)	H8A—C8—H8C	109.5
C6—N1—Cd1	123.1 (2)	H8B—C8—H8C	109.5
C7—N2—C1	109.0 (3)	C10—C9—C14	120.6 (4)
C7—N2—H2	125.5	C10—C9—N3	129.7 (4)
C1—N2—H2	125.5	C14—C9—N3	109.7 (3)
C15—N3—C9	105.3 (3)	C11—C10—C9	116.6 (4)
C15—N3—Cd1	132.3 (3)	C11—C10—H10	121.7
C9—N3—Cd1	122.3 (2)	C9—C10—H10	121.7
C15—N4—C14	109.1 (3)	C10—C11—C12	121.9 (4)
C15—N4—H4	125.4	C10—C11—H11	119.1
C14—N4—H4	125.4	C12—C11—H11	119.1
N2—C1—C2	132.2 (4)	C13—C12—C11	122.1 (4)
N2—C1—C6	105.3 (3)	C13—C12—H12	119.0
C2—C1—C6	122.5 (4)	C11—C12—H12	119.0
C3—C2—C1	116.3 (4)	C12—C13—C14	116.5 (4)
C3—C2—H2A	121.8	C12—C13—H13	121.8
C1—C2—H2A	121.8	C14—C13—H13	121.8
C2—C3—C4	122.2 (4)	C13—C14—N4	133.4 (4)
C2—C3—H3	118.9	C13—C14—C9	122.3 (4)
C4—C3—H3	118.9	N4—C14—C9	104.2 (3)
C5—C4—C3	121.0 (5)	N3—C15—N4	111.7 (3)
C5—C4—H4A	119.5	N3—C15—C16	125.5 (4)
C3—C4—H4A	119.5	N4—C15—C16	122.8 (3)
C4—C5—C6	117.5 (4)	C15—C16—H16A	109.5
C4—C5—H5	121.2	C15—C16—H16B	109.5
C6—C5—H5	121.2	H16A—C16—H16B	109.5
N1—C6—C5	130.8 (4)	C15—C16—H16C	109.5
N1—C6—C1	108.8 (3)	H16A—C16—H16C	109.5
C5—C6—C1	120.4 (4)	H16B—C16—H16C	109.5
N1—C7—N2	110.8 (3)	N3—Cd1—N1	106.04 (12)
N1—C7—C8	125.9 (4)	N3—Cd1—Br1	109.95 (9)
N2—C7—C8	123.3 (4)	N1—Cd1—Br1	117.77 (9)
C7—C8—H8A	109.5	N3—Cd1—Br2	107.95 (9)
C7—C8—H8B	109.5	N1—Cd1—Br2	105.40 (9)
H8A—C8—H8B	109.5	Br1—Cd1—Br2	109.28 (2)
			
C7—N2—C1—C2	178.9 (4)	C9—C10—C11—C12	1.2 (6)
C7—N2—C1—C6	0.1 (4)	C10—C11—C12—C13	−2.5 (7)
N2—C1—C2—C3	−177.2 (4)	C11—C12—C13—C14	1.1 (6)
C6—C1—C2—C3	1.3 (6)	C12—C13—C14—N4	−179.9 (4)
C1—C2—C3—C4	0.1 (7)	C12—C13—C14—C9	1.4 (6)
C2—C3—C4—C5	−1.5 (7)	C15—N4—C14—C13	−177.7 (4)
C3—C4—C5—C6	1.5 (7)	C15—N4—C14—C9	1.2 (4)
C7—N1—C6—C5	−177.1 (4)	C10—C9—C14—C13	−2.7 (6)
Cd1—N1—C6—C5	11.3 (6)	N3—C9—C14—C13	177.7 (3)
C7—N1—C6—C1	0.9 (4)	C10—C9—C14—N4	178.3 (3)
Cd1—N1—C6—C1	−170.7 (2)	N3—C9—C14—N4	−1.4 (4)
C4—C5—C6—N1	177.6 (4)	C9—N3—C15—N4	−0.2 (4)
C4—C5—C6—C1	−0.2 (6)	Cd1—N3—C15—N4	175.6 (2)
N2—C1—C6—N1	−0.6 (4)	C9—N3—C15—C16	−179.7 (4)
C2—C1—C6—N1	−179.5 (3)	Cd1—N3—C15—C16	−3.9 (6)
N2—C1—C6—C5	177.6 (4)	C14—N4—C15—N3	−0.6 (4)
C2—C1—C6—C5	−1.3 (6)	C14—N4—C15—C16	178.9 (4)
C6—N1—C7—N2	−0.9 (4)	C15—N3—Cd1—N1	−4.6 (3)
Cd1—N1—C7—N2	169.9 (2)	C9—N3—Cd1—N1	170.6 (2)
C6—N1—C7—C8	177.7 (4)	C15—N3—Cd1—Br1	123.7 (3)
Cd1—N1—C7—C8	−11.5 (6)	C9—N3—Cd1—Br1	−61.0 (3)
C1—N2—C7—N1	0.5 (4)	C15—N3—Cd1—Br2	−117.1 (3)
C1—N2—C7—C8	−178.1 (4)	C9—N3—Cd1—Br2	58.1 (3)
C15—N3—C9—C10	−178.6 (4)	C7—N1—Cd1—N3	112.4 (3)
Cd1—N3—C9—C10	5.1 (5)	C6—N1—Cd1—N3	−78.2 (3)
C15—N3—C9—C14	1.0 (4)	C7—N1—Cd1—Br1	−11.1 (4)
Cd1—N3—C9—C14	−175.3 (2)	C6—N1—Cd1—Br1	158.3 (2)
C14—C9—C10—C11	1.3 (5)	C7—N1—Cd1—Br2	−133.3 (3)
N3—C9—C10—C11	−179.1 (3)	C6—N1—Cd1—Br2	36.2 (3)

### Hydrogen-bond geometry (Å, º)

**Table:**

*D*—H···*A*	*D*—H	H···*A*	*D*···*A*	*D*—H···*A*
N2—H2···Br1^i^	0.86	2.88	3.495 (4)	130
N4—H4···Br2^ii^	0.86	2.77	3.563 (4)	155

Symmetry codes: (i) −*x*+1, *y*−1/2, −*z*+3/2; (ii) −*x*, *y*−1/2, −*z*+3/2.
